# Multiple-breath washout at 12 months of age reveals lung function impairments in preterm infants with bronchopulmonary dysplasia

**DOI:** 10.1186/s40348-026-00248-x

**Published:** 2026-07-28

**Authors:** Isabell Ricklefs, Elisa Grützner, Hannah Wedman, Mats Ingmar Fortmann, Désirée Lasserre, Nikolas Jakobs, Friederike Pagel, Fumi Sugihara, Wolfgang Göpel, Folke Christina Brinkmann, Matthias Volkmar Kopp, Mirjam Stahl, Christoph Härtel, Julia Pagel

**Affiliations:** 1 Department of Pediatrics, University of Lübeck, Campus Lübeck Ratzeburger Allee 160, Lübeck, 23538 Germany; 2 Airway Research Center North, German Center for Lung Research, Lübeck, Germany; 3 Department of Obstetrics and Gynocology, Altötting Hospital, Altötting, Germany; 4 Department of Pediatrics, Agaplesion Klinikums Hagen, Hagen, Germany; 5 Detlef Zillikens Clinician Scientist Academy Lübeck, Lübeck, Germany; 6 University Children’s Hospital, University of Würzburg, Würzburg, Germany; 7 Department of Pediatrics, Inselspital, Bern University Hospital, Bern, Switzerland; 8 Department of Pediatric Respiratory Medicine, Immunology and Critical Care Medicine, Charité – Universitätsmedizin Berlin, Berlin, Germany; 9 German Center for Lung Research, associated partner site, Berlin, Germany; 10 German Center for Infection Research (DZIF), partner site Hamburg- Lübeck-Borstel-Riems, Lübeck, Germany; 11 Department of Pediatrics, University Hospital Hamburg-Eppendorf, Hamburg, Germany

**Keywords:** Bronchopulmonary dysplasia, Multiple breath washout, Lung function

## Abstract

**Background:**

Bronchopulmonary dysplasia (BPD) is a major complication of preterm birth and may lead to long-term respiratory morbidity. Early measurements of impaired lung function are needed to guide the development of preventive strategies. This study aimed to evaluate the feasibility and potential utility of MBW-derived parameters for detecting early lung function impairment in preterm infants at a corrected age of 12 months.

**Methods:**

We conducted a single-centre study of preterm infants at 12 months’ corrected age using sulfur hexafluoride MBW during spontaneous sleep. Tidal Breathing Flow Volume Loops (TBFVL) and MBW were used to assess respiratory parameters including lung clearance index (LCI). This was complemented by parental questionnaires on infant health. The cohort was stratified into three groups based on BPD diagnosis: (I) severe BPD (oxygen requirement at 28 days and respiratory support at 36 weeks’ postmenstrual age), (II) mild BPD (oxygen requirement at 28 days but no oxygen or respiratory support at 36 weeks’ postmenstrual age ) and (III) no BPD.

**Results:**

Reliable TBFVL and MBW measurements during spontaneous sleep were obtained in 45 of 112 infants (40%) including 8 infants with severe BPD and 7 infants with mild BPD. Infants with BPD exhibited higher LCI values (7.0 vs. 6.5, *p* = 0.018) and lower ratios of tidal volume to functional residual capacity (0.40 vs. 0.51, *p* = 0.022) compared to infants without BPD. Infants with severe BPD had higher LCI values than those without BPD (7.1 vs. 6.5, *p* = 0.022). LCI was positively correlated with the duration of continuous positive airway pressure (CPAP) support (Pearson correlation coefficient *r* = 0.476, *p* = 0.001), oxygen supplementation (*r* = 0.444, *p* = 0.002) and antibiotic treatment (*r* = 0.435, *p* = 0.003), respectively. Higher LCI values were also associated with an increased rate of hospital readmissions due to respiratory infections.

**Conclusions:**

Despite limited feasibility, MBW measurements obtained during spontaneous sleep at 12 months’ corrected age revealed impaired lung function in preterm infants with BPD. LCI correlated with the duration of antibiotic treatment, CPAP support, and oxygen supplementation all of which represent potentially modifiable clinical factors. These findings suggest that LCI may serve as a promising early lung function parameter for long-term respiratory vulnerability.

**Supplementary Information:**

The online version contains supplementary material available at 10.1186/s40348-026-00248-x.

## Introduction

Bronchopulmonary dysplasia (BPD) remains one of the most common complications of preterm birth despite considerable progress in neonatal care, including surfactant treatment, antenatal corticosteroids, and improved respiratory support strategies. BPD occurs in 15–40% of very low birth weight infants (birth weight < 1500 g) and represents a key factor of long-term respiratory morbidity in this vulnerable population [1–3].

Children and adults born with a history of BPD frequently exhibit persistent respiratory symptoms and lung function impairment across the lifespan [1, 4–6]. Different clinical definitions of BPD exist. In the present study, BPD was classified according to the 2001 NICHD consensus definition. Irrespective of the definition used, BPD is associated with an increased risk for the clinical phenotype of “chronic pulmonary insufficiency after prematurity” [7] which is characterized by increased susceptibility to respiratory infections and both obstructive and restrictive lung function deficits [8–13]. Long-term follow-up studies have demonstrated that individuals with previous BPD diagnosis are at increased risk for “physician-diagnosed asthma” [14, 15], impaired exercise capacity [5] and a more rapid decline in lung function over time during adulthood [15–20]. In addition, BPD is associated with long-term adverse neurodevelopmental and physical outcomes [21–23].

Given the substantial long-term burden of respiratory diseases after preterm birth, there is an urgent need to identify early parameters of lung function impairment to be evaluated as clinical readouts in order to guide individualized prevention and intervention strategies. However, the interpretation of existing data for lung function development in infancy remains challenging due to the use of different techniques and missing reference values [24–26]. Multiple-breath washout (MBW) has first been described approximately 70 years ago [27], but has emerged as a promising method for assessing early lung function in infants and young children. MBW allows the evaluation of ventilation inhomogeneity and small airway dysfunction by calculating the lung clearance index (LCI), which reflects the efficiency of gas mixing within the lungs [28–31]. Previous studies using tidal breathing flow-volume loops (TBFVL) and MBW in BPD patients have reported heterogenous findings during the neonatal period. For example, a lower time to peak tidal expiratory flow to expiratory time ratio has been described in infants receiving oxygen therapy at 36 weeks’ postmenstrual age (no or mild BPD versus moderate or severe BPD) [32]. The same study reported increased respiratory rates with increasing BPD disease severity and lower minute ventilation per bodyweight for infants with BPD (all severity groups) versus healthy preterm and term neonates [32]. These early alterations in respiratory mechanic may contribute to lung function impairment observed later in childhood and adolescence [26, 33]. Despite increasing use of MBW in pediatric respiratory research, data on MBW-derived lung function parameters in preterm infants beyond the neonatal period remain limited. In particular, the feasibility and potential utility of MBW measurements in spontaneously sleeping preterm infants for detecting early lung function impairment during the first year of life have not been extensively studied. Therefore, we performed lung function testing using TBFVL and sulfur hexafluoride (SF6) MBW in preterm infants at 12 months’ corrected age. We divided our cohort into three categories based on the BPD diagnosis: (I) severe BPD (respiratory support at 36 weeks’ postmenstrual age), (II) mild BPD (oxygen need at 28 days), (III) no BPD. The aim of the study was to test feasibility of TBFVL and MBW measurements during spontaneous sleep in premature infants at 12 months’ corrected age. Additionally, we hypothesized that preterm infants with BPD exhibit ventilation inhomogeneities, reflected by increased LCI values. Furthermore, we wanted to explore associations between lung function parameters, duration of neonatal respiratory support, and respiratory morbidity during the first year of life.

## Methods

### Study cohort and ethics

We established a follow-up examination protocol for former preterm infants at a corrected age of 12 months (+/- 3 weeks) as part of the Immunoregulation of the Newborn (IRoN) study [34] in the Department of Pediatrics at the University Hospital of Lübeck. During the time from May 2017 until September 2019, all preterm infants with a gestational age of ≥ 23 + 0 and < 37 + 0 weeks were offered participation for follow-up examination. Informed written consent was obtained from both parents or legal representatives. The study was approved by the local committee on research in human subjects at the University of Lübeck (IRoN AZ 15–304). The follow-up examination consisted of a parent report on development and morbidities (e.g. infections) during the first year of life, a physical examination as well as lung function testing using the TBFVL and MBW technique.

BPD was defined by the National Institute of Child Health and Human Development consensus definition of 2001 [35]. BPD definition was based on two timepoints: 28 days of life and 36 weeks’ postmenstrual age. Mild BPD was defined as oxygen requirement at 28 days of life, but no need of oxygen or ventilation support at 36 weeks’ postmenstrual age. Moderate BPD was defined as oxygen requirement at 28 days of life and FiO2 < 0.3 at 36 weeks’ postmenstrual age. However, in our cohort no infants fulfilled the criteria of moderate BPD. Severe BPD was defined as oxygen requirement at 28 days of life and ≥ FiO2 0.3 or requirement of any respiratory support at 36 weeks’ postmenstrual age. Infants without supplemental oxygen or respiratory support at 36 weeks’ postmenstrual age were classified as breathing room air, corresponding to an FiO₂ of 0.21. In infants receiving supplemental oxygen, oxygen was administered via high-flow nasal cannula, allowing documentation of the delivered FiO₂. For the present analysis, infants were categorized into three groups: severe BPD, mild BPD, and no BPD. Gestational age was calculated from the best obstetric estimate based on early prenatal ultrasound and obstetric examination [36].

### Outcome measures

#### Lung function testing

Lung function measurements were performed according to a standardized protocol throughout the study period. The examination was performed in a quiet and darkened room, typically around midday and preferably after feeding. Infants remained in a stroller or infant carrier familiar to the family whenever possible. Infants were placed in a supine position as previously described [37, 38] during spontaneous sleep. All infants were breathing spontaneously without need for extra oxygen. Parents were continuously present during the examination and played an important role in calming and preparing the infant for sleep. Measurements were performed using a face mask and only one study visit was scheduled per day to align the timing of the measurement with the infant’s natural sleep behavior and to allow sufficient time for spontaneous sleep onset and completion of the investigation.

TBFVL as well as MBW with 4% SF_6_ were performed using the EXHALYZER D (EcoMedics AG, Duernten, Switzerland). TBFVL was always performed before MBW measurement. Calibration of the device was performed following the standard operating procedure provided by the German Centre for Lung Research [37, 38]. TBFVL was used to measure respiratory rate (RR = 1/min), tidal volume (VT) and minute ventilation (MV) while MBW was used to determine LCI as the ratio of cumulative exhaled volume (CEV) / FRC among other parameters. We used LCI 2.5% measuring the number of lung turnovers required for the SF_6_ gas to be washed out to 2.5% of its initial starting concentration. During the time of this investigation, the Exhalyzer D Software had been updated from Spiroware 2.0 to Spiroware 3.2.1. *n* = 33 measurements were performed with Spiroware 2.0, *n* = 12 measurements were performed with Spiroware 3.2.1. and reanalysed in Spiroware 3.3.

#### Parental questionnaire

During the follow-up examination, parents responded to a questionnaire that was developed by our study group and was not a standardized instrument. A selection of the items of the questionnaire relevant to this publication can be found in Supplemental Fig. 1. Parents were asked how many upper respiratory tract infections the child had in the past year. We used the 75th percentile of the total number of upper respiratory tract infections as a cut-off value to identify infants with a comparatively high burden of recurrent respiratory morbidity.

### Statistical methods

Clinical characteristics and lung function results are presented as mean values ± standard deviations (SD), and comparisons were performed using a t-test or ANOVA for normally distributed data. For non-normally distributed data, the non-parametric Mann–Whitney U test was used. For categorical variables, data are presented as absolute numbers and percentages of the total study population (%). Comparisons between groups were performed using the chi-squared test. Correlation between LCI and clinical parameters (gestational age, birth weight, duration of CPAP, oxygen supplementation and antibiotic treatment) was calculated by using the Pearson correlation coefficient. Statistical analysis was performed using IBM SPSS Statistics 28.0 (Armonk, NY, USA). A *p*-value below 0.05 was considered statistically significant. Due to the exploratory nature of this single-centre observational study, no formal sample size calculation was performed prior to study initiation. Reported *p*-values in Tables 1, 2 and 3 are descriptive and not corrected for multiple testing.

## Results

### Clinical characteristics

**Fig. 1:**
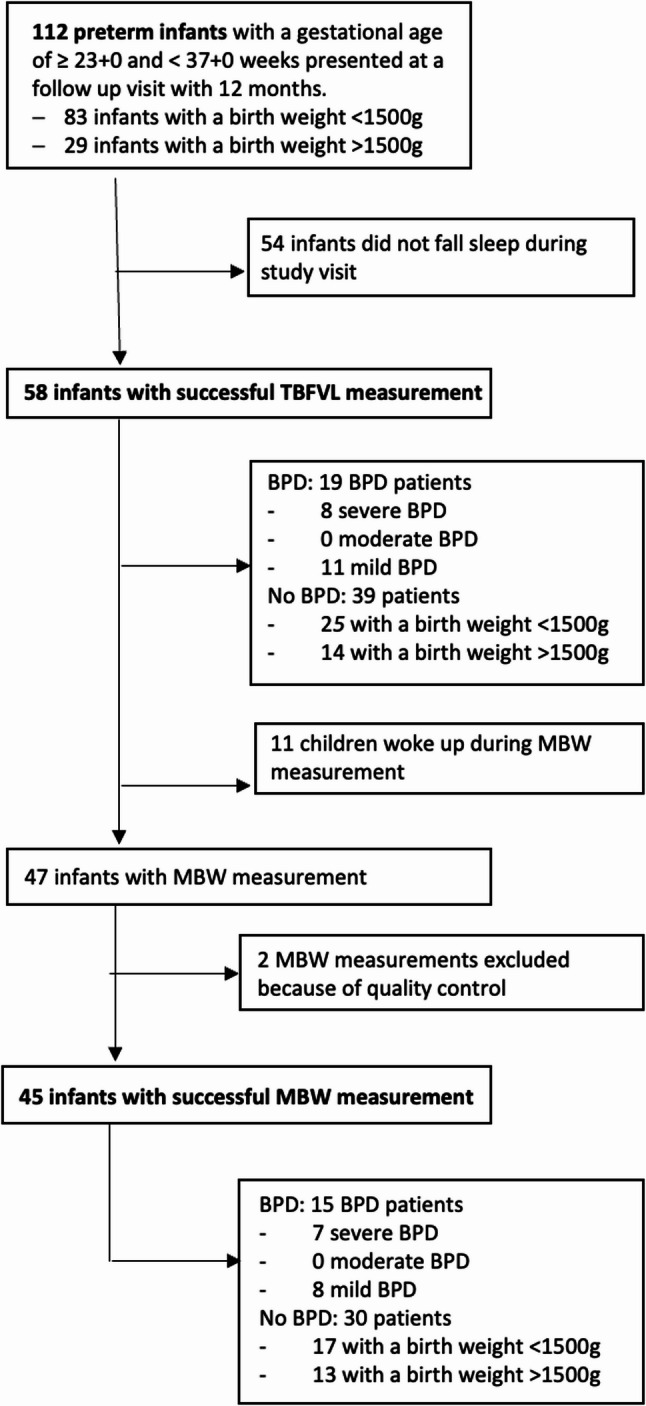
Flow chart of IRoN study participants included in the analysis

Between May 2017 and September 2019, we conducted study visits for 112 preterm infants of the IRoN cohort at a corrected age of 12 months (Table 1) (Fig. 1). Lung function testing using TBFVL and MBW measurements were planned for all participants. TBFVL measurements were successfully performed in 58 infants (52%), whereas 54 infants did not fall asleep, so that we could not examine any lung function in those infants. MBW measurement was always performed after TBFVL. 11 infants woke up during MBW measurement and two measurements were excluded after quality control. Thus, MBW measurements were reliably obtained in 45 infants (40%) (Fig. 1). Patients with successful TBFVL and MBW measurements did not differ in relevant clinical parameters from those in whom lung function could not be performed successfully with the exception of duration of CPAP therapy and incidence of late onset sepsis (LOS) (Table 1). CPAP duration was longer and the rate of LOS higher in the group of patients with successful TBFVL.

**Table 1:** Clinical characteristics of preterm infants with and without successful TBFVL and MBW measurements

	All patients *n* = 112	Patients without TBFVL, *n* = 54	Patients with TBFVL, *n* = 58	*p*-value*	Patients without MBW, *n* = 67	Patients with MBW, *n* = 45	*p*-value**
GA (w)	29.8 ± 3.2	30.3 ± 3.2	29.3 ± 3.2	0.113	30.0 ± 3.2	29.5 ± 3.3	0.413
BW (g)	1354 ± 594	1382 ± 551	1327 ± 635	0.627	1340 ± 540	1374 ± 673	0.771
Sex - male	62 (55%)	28 (52%)	34 (59%)	0.472	35 (52%)	27 (60%)	0.418
Caesarean section	96 (86%)	48 (89%)	48 (83%)	0.100	60 (90%)	36 (80%)	0.157
Surfactant	59 (53%)	24 (44%)	35 (60%)	0.112	32 (48%)	27 (60%)	0.160
Mechanical ventilation (d)	2.4 ± 7.3	1.8 ± 7.9	3.0 ± 6.8	0.401	2.2 ± 8.2	2.7 ± 5.7	0.733
CPAP (d)	31.0 ± 31	24.9 ± 28.0	36.7 ± 32.7	0.043	28.0 ± 28.8	35.4 ± 33.8	0.220
O2 (d)	21.0 ± 34.6	15.2 ± 32.5	26.3 ± 35.9	0.091	17.1 ± 33.4	26.6 ± 35.9	0.156
EOS	24 (21%)	8 (15%)	16 (28%)	0.090	9 (13%)	15 (33%)	0.110
LOS	24 (21%)	7 (13%)	17 (29%)	0.031	11 (16%)	13 (29%)	0.125
ATB (d)	22.9 ± 35.5	19.3 ± 36.4	26.2 ± 34.4	0.311	20.3 ± 34.7	26.7 ± 36.6	0.349
URTI (#)	3.6 ± 3.2	3.9 ± 3.9	3.3 ± 3.3	0.295	3.7 ± 3.4	3.5 ± 2.9	0.777
Bronchitis (#)	1.1 ± 2.4	0.9 ± 1.6	1.2 ± 3.0	0.513	0.9 ± 1.5	1.4 ± 3.3	0.218
Hospital re-admission^§^	14 (12.5%)	5 (9%)	9 (15%)	0.374	8 (12%)	6 (13%)	0.752
Body length 12 M (cm)	74.7 ± 3.7	75.3 ± 3.0	74.1 ± 4.2	0.077	74.8 ± 3.5	74.5 ± 4.0	0.657
Body weight 12 M (kg)	9.31 ± 1.29	9.32 ± 1.27	9.29 ± 1.31	0.883	9.25 ± 1.24	9.39 ± 1.36	0.590
BPD	31 (28%)	12 (22%)	19 (33%)	0.259	16 (24%)	15 (33%)	0.273
Severe BPD	14 (45%)	3 (25%)	11 (58%)	0.073	6 (27%)	8 (53%)	0.376

Data are described as mean ± standard deviation or *n* (%)

*Abbreviations*: *TBFVL* Tidal breathing flow-volume loops, *MBW* Multiple breath washout, *GA (w)* Gestational age in weeks, *BW (g)* Birth weight in grams, *CPAP (d)* Duration of CPAP ventilation in days, *O2 (d)* Duration of oxygen supplementation in days, *EOS* Early onset sepsis, *LOS* Late onset sepsis (clinically diagnosed or blood culture positive), *ATB (d)* Duration of intravenous antibiotic treatment in days, *URTI (#)* Number of upper respiratory tract infections, *§ H*ospital re-admission due to respiratory symptoms, *12 M* 12 months, *BPD* Bronchopulmonary dysplasia

*P*- values are descriptive and not corrected for multiple testing

*comparing patients with successful TBFVL measurements to patients without successful lung function

**comparing patients with successful MBW measurements to patients without MBW measurements

We included infants in the following analyses who completed TBFVL and MBW (*n* = 45) and excluded infants with incomplete results because they did not fall asleep, did not stay asleep for the duration of the MBW measurement or MBW measurements did not pass quality control. In the final population, 15 out of 45 (30%) patients were diagnosed with a BPD (8 patients with severe BPD and 7 patients with mild BPD). No infants in our cohort fulfilled the criteria for a moderate BPD. Patients with BPD had a lower gestational age and required mechanical ventilation, CPAP ventilation and oxygen supplementation for a longer period compared to infants without BPD (Table 2). BPD patients developed a LOS more often and needed a longer period of antibiotic treatment. During the first 12 months, 5 infants with BPD were readmitted to the hospital due to respiratory symptoms as compared to no infant without BPD. We found the same results when reducing the group of infants without BPD to the ones with a birth weight < 1500 g (Supplemental Table 1). Infants with severe BPD required oxygen and CPAP treatment for longer periods and suffered from upper respiratory tract infections more often compared to patients with mild BPD (Table 2). None of the preterm infants still required mechanical ventilation or oxygen supply at a corrected age of 12 months.

**Table 2:** Clinical characteristics of preterm infants with successful MBW measurements stratified by BPD status and severity

	No BPD *n* = 30	BPD *n* = 15	*p*-value *	Mild BPD *n* = 7	Severe BPD *n* = 8	*p*-value **
GA (w)	31.1 ± 2.5	26.2 ± 1.7	< 0.001	26.3 ± 1.2	26.1 ± 2.1	0.827
BW (g)	1639 ± 649	845 ± 319	< 0.001	873 ± 218	820 ± 401	0.762
Sex - male	17 (57%)	10 (67%)	0.519	3 (43%)	7 (88%)	0.067
Caesarean section	23 (77%)	13 (87%)	0.429	7 (100%)	6 (75%)	0.155
Surfactant	13 (43%)	14 (93%)	0.002	6 (86%)	8 (100%)	0.268
Mechanical ventilation (d)	0.13 ± 0.51	7.8 ± 7.65	0.002	6.1 ± 8.5	9.3 ± 7.0	0.453
CPAP (d)	17.3 ± 16.3	71.3 ± 31	< 0.001	51.6 ± 12	90.8 ± 32.5	0.015
O2 (d)	5.2 ± 7.57	69.47 ± 31.4	< 0.001	45.1 ± 7.2	90.8 ± 28.6	0.001
EOS	9 (31%)	6 (10%)	0.552	1 (14%)	5 (63%)	0.057
LOS	3 (10%)	10 (67%)	< 0.001	3 (43%)	7 (88%)	0.067
ATB (d)	10.7 ± 8	58.8 ± 49.2	0.002	37.8 ± 17	77.1 ± 61.5	0.127
URTI (#)	3.8 ± 3.3	2.9 ± 1.8	0.351	1.6 ± 1	4.1 ± 1.5	0.002
Bronchitis (#)	1.67 ± 3.9	0.93 ± 1.4	0.490	0.4 ± 0.5	1.4 ± 1.8	0.198
Hospital re-admission^§^	0	5 (35%)	< 0.001	1 (14%)	4 (50%)	0.143
Body length 12 M (cm)	75.2 ± 3.2	73.3 ± 4.9	0.185	74 ± 4.3	72.6 ± 5.6	0.609
Body weight 12 M (kg)	9.67 ± 1.33	8.84 ± 1.28	0.054	9.17 ± 1.40	8.54 ± 1.18	0.360

Data are described as mean ± standard deviation or *n* (%)

*Abbreviations*: *GA (w)* Gestational age in weeks, *BW (g)* Birth weight (grams), *CPAP (d)* Duration of CPAP ventilation in days, *O2 (d)* Duration of oxygen supplementation in days, *EOS* Early onset sepsis, *LOS* Late onset sepsis (clinically diagnosed or blood culture positive), *ATB (d)* Duration of intravenous antibiotic treatment in days, *URTI (#)* Number of upper respiratory tract infections, *§ H*ospital re-admission due to respiratory symptoms, *12 M* 12 months, *BPD* Bronchopulmonary dysplasia

*P*- values are descriptive and not corrected for multiple testing

*comparison of patients with BPD to all patients without BPD

**comparison mild BPD and severe BPD

### Lung function parameters at 12 months corrected age

**Fig. 2:**
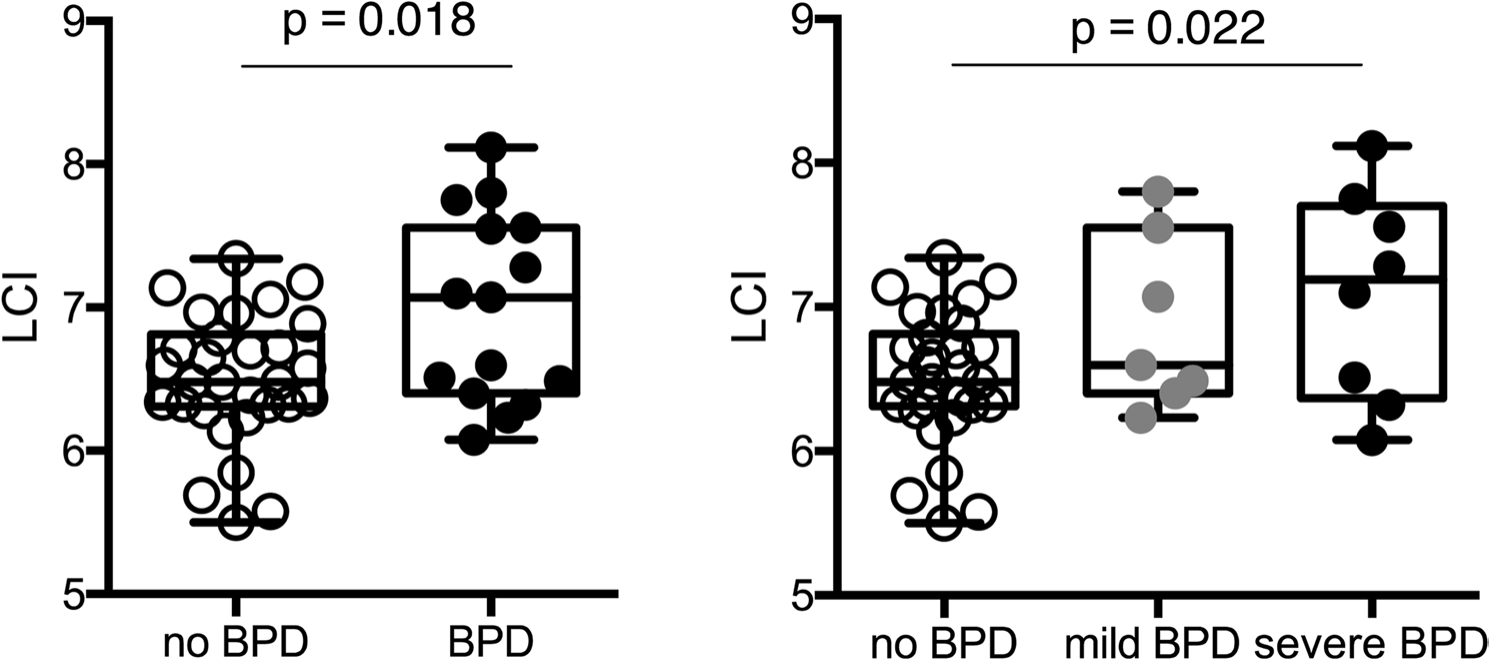
LCI values in preterm infants stratified by BPD status and severity. Infants with BPD had significantly higher LCI values than infants without BPD. Infants with severe BPD also had higher LCI values than infants without BPD. Mean LCI values were compared using an unpaired t-test for comparisons between two groups and one-way ANOVA for comparisons between three groups

Lung function parameters derived from TBFVL and MBW measurements are summarized in Table 3. Infants with the diagnosis of BPD had a higher LCI at 12 corrected months compared to infants without BPD (7.0 vs. 6.5, *p* = 0.018), indicating increased ventilation inhomogeneity in BPD (Fig. 2; Table 3). In addition, the VT/FRC ratio was significantly lower in infants with BPD (0.40 vs. 0.51, *p* = 0.022). When stratified by BPD severity, infants with severe BPD exhibited higher LCI values (7.1 vs. 6.5, *p*-value: 0.022) compared to infants without BPD (Fig. 2). No statistically significant difference in the MBW parameters was observed between infants with severe BPD and infants with mild BPD (Table 3). Tidal volume and minute ventilation were lower in infants with BPD compared to infants without BPD, while this difference was not confirmed after adjustment for body weight (Table 3). Of note, the findings were consistent when comparing infants with BPD to the subgroup of infants without BPD and a birth weight < 1500 g (Supplemental Table 2).

**Table 3:** TBFVL and MBW results in preterm infants stratified by BPD status and severity

TBFVL, *n* = 58	No BPD *n* = 39	BPD *n* = 19	*p*-value *	Mild BPD *n* = 8	Severe BPD *n* = 11	*p*-value **
RR (/min)	27 ± 5	28 ± 9	0.347	27 ± 3	29 ± 12	0.531
MV (ml/min)	2668 ± 410	2420 ± 363	0.029	2436 ± 467	2409 ± 292	0.872
MV/weight (ml/min*kg)	285 ± 40	279 ± 38	0.613	270 ± 45	286 ± 33	0.385
VT (ml)	103 ± 17	91 ± 21	0.022	92 ± 16	90 ± 24	0.858
VT/weight (ml/kg)	11 ± 1.7	10 ± 2.1	0.262	10 ± 2.0	11 ± 2.3	0.811

MBW, *n* = 45	No BPD *n* = 30	BPD *n* = 15	*p*-value *	Mild BPD *n* = 7	Severe BPD *n* = 8	*p*-value **
FRC (l)	0.23 ± 0.08	0.23 ± 0.06	0.820	0.23 ± 0.03	0.23 ± 0.08	0.971
VT/FRC	0.51 ± 0.15	0.40 ± 0.12	0.022	0.40 ± 0.07	0.40 ± 0.16	0.991
LCI	6.5 ± 0.45	7.0 ± 0.66	0.018	6.9 ± 0.6	7.1 ± 0.72	0.553

Data are described as mean ± standard deviation or *n* (%)

*Abbreviations*: *BPD* Bronchopulmonary dysplasia, *TBFVL* Tidal breathing flow-volume loops, *MBW* Multiple breath washout, *RR* Respiratory rate, *MV* Minute volume, *VT* Tidal volume, *FRC* Functional residual capacity, *LCI* Lung clearance index, *t *test

*P*- values are descriptive and not corrected for multiple testing

*comparison of patients with BPD to all patients without BPD

**comparison mild BPD and severe BPD

### Association between LCI and neonatal clinical factors

**Fig. 3:**
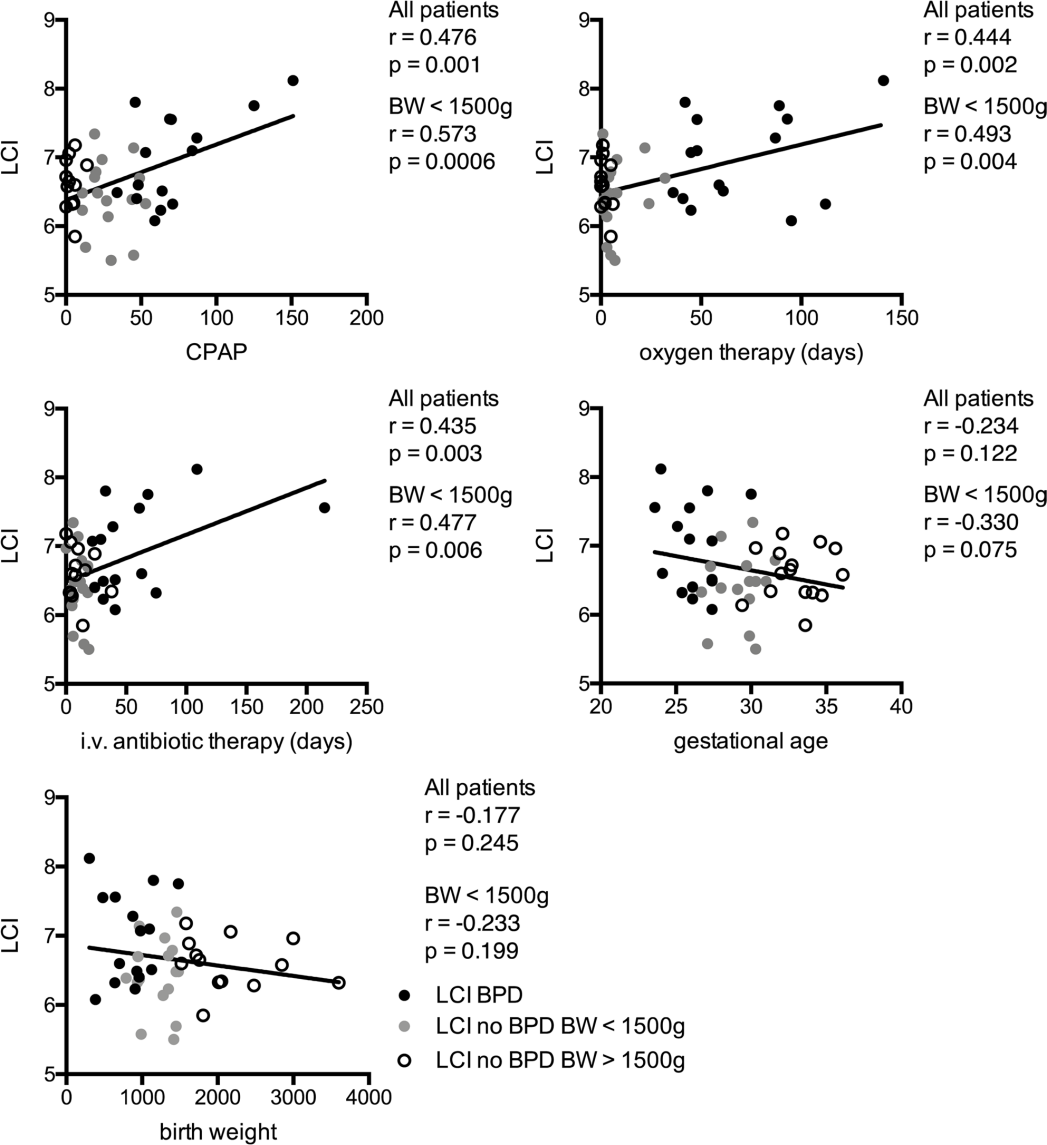
Correlations between LCI and neonatal clinical parameters. LCI was positively correlated with the duration of CPAP support, oxygen supplementation, and intravenous antibiotic treatment, whereas gestational age and birth weight were not associated with LCI. Pearson correlation analyses are shown for the overall cohort and for the subgroup of infants with a birth weight < 1500 g

**Fig. 4:**
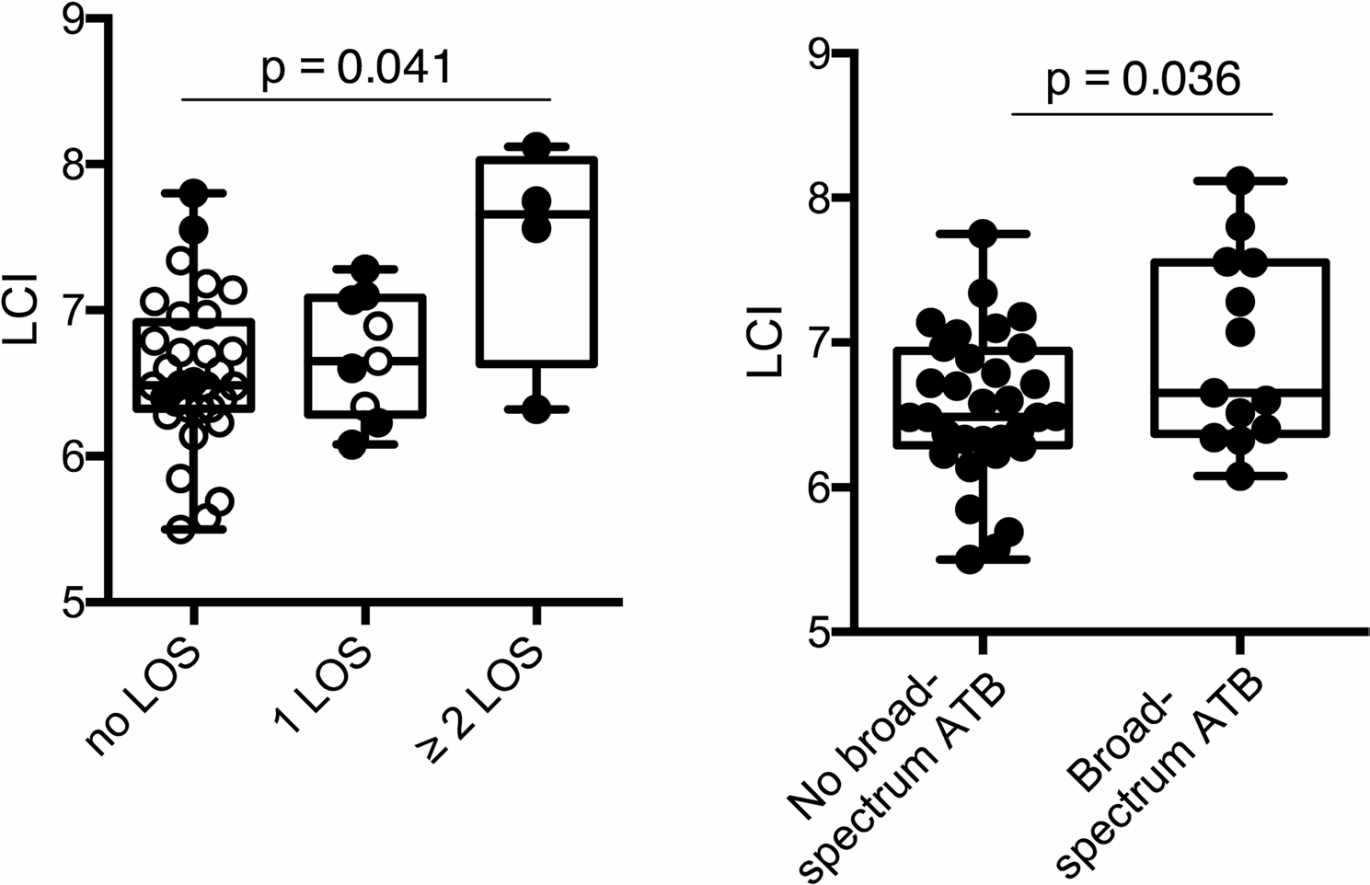
LCI values according to late-onset sepsis (LOS) episodes and broad-spectrum antibiotic treatment. LCI values were compared between infants with no LOS, one LOS episode, and two or more LOS episodes. LOS was defined as either clinically diagnosed LOS or blood culture-positive LOS. Differences between LOS groups were assessed using one-way ANOVA. LCI values were also compared between infants who required broad-spectrum antibiotic treatment with meropenem, linezolid, or cefotaxime and infants without exposure to broad-spectrum antibiotics. Groups were compared using an unpaired t-test

To explore potential determinants of lung function impairment, we assessed correlations between LCI and selected neonatal clinical parameters (Fig. 3). LCI showed a positive correlation with the duration of CPAP support (Pearson correlation coefficient *r* = 0.476, *p* = 0.001), duration of oxygen supplementation (*r* = 0.444, *p* = 0.002) and duration of antibiotic treatment (*r* = 0.435, *p* = 0.003), respectively. Of note, the LCI was neither correlated with gestational age (*r*=-0.234 *p* = 0.122) nor birth weight (*r*=-0.177, *p* = 0.245) (Fig. 3). We observed the same correlations in a subgroup analysis only including patients with a birth weight < 1500 g. In line with the observed associations of antibiotic treatment duration with the LCI, we found that infants with recurrent LOS episodes showed higher LCI values (Fig. 4). Furthermore, infants who required broad-spectrum antibiotic treatment (meropenem, linezolid, cefotaxime) exhibited higher LCI values (*n* = 13 LCI 6.95) compared with infants without exposure to broad-spectrum antibiotics (*n* = 32 LCI 6.55, *p* = 0.036) (Fig. 4).

**Fig. 5:**
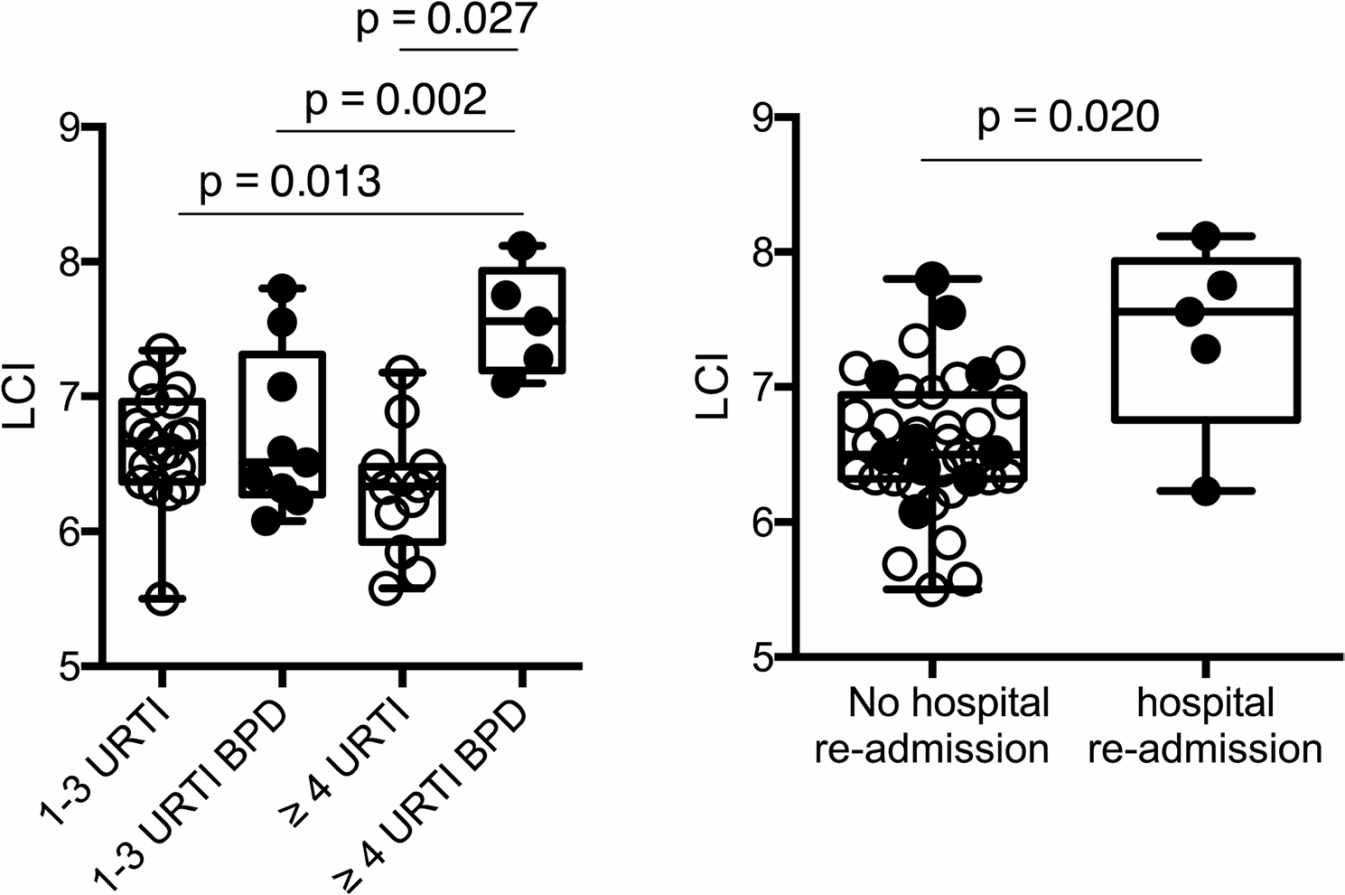
LCI values according to respiratory morbidity during the first year of life. Infants with BPD are shown as filled dots, and infants without BPD as open dots. Infants with BPD and four or more upper respiratory tract infections (URTIs; *n* = 5) had higher LCI values than infants with BPD and fewer than four URTIs, as well as infants without BPD irrespective of the number of URTIs. Infants readmitted to hospital due to respiratory symptoms (*n* = 5) had higher LCI values than infants without respiratory hospital readmission. Comparisons between the four URTI/BPD groups were performed using one-way ANOVA followed by Games–Howell post hoc analysis. LCI values according to hospital readmission status were compared using the Mann–Whitney U test

Respiratory morbidity during the first year of life was assessed using parental reports. A cut-off of four or more upper respiratory tract infections (URTIs), corresponding to the 75th percentile in our cohort, was used to define a high burden of recurrent respiratory morbidity. Infants with BPD and four or more URTIs during the first year of life (*n* = 5) had significantly higher LCI values than infants with BPD and fewer URTIs, as well as infants without BPD irrespective of the number of reported URTIs (Fig. 5). All infants with BPD and four or more URTIs belonged to the severe BPD subgroup and had required longer durations of CPAP support and oxygen supplementation. They also developed bronchitis more frequently and were more often readmitted to the hospital due to respiratory symptoms during the first year of life (Supplemental Table 3). Among infants without BPD, those with four or more URTIs had a slightly lower gestational age and more frequently developed bronchitis during the first year of life (Supplemental Table 3). Consistent with these findings, infants who were readmitted to the hospital due to respiratory symptoms (*n* = 5) had higher LCI values than those without respiratory hospital readmission (7.4 vs. 6.5; *p* = 0.02; Fig. 5). Most infants requiring hospital readmission had severe BPD. Additional clinical data are provided in Supplemental Table 4.

## Discussion

In this single-centre study of preterm infants at 12 corrected months of age we demonstrated the feasibility of MBW assessments during spontaneous sleep in a subset of infants and revealed lung function abnormalities in those with a history of BPD. Infants with BPD showed significantly higher LCI values compared to preterm infants without BPD, indicating increased ventilation inhomogeneity. LCI values were highest in infants with severe BPD and correlated positively with the duration of CPAP ventilation and oxygen supplementation. Intriguingly, LCI values were also linked to the duration of intravenous antibiotic treatment, number of LOS epsiodes, need of reserve antibiotic treatment and to respiratory morbidity during the first year of life.

Our findings are consistent with previous studies showing that preterm birth and BPD are associated with long-term alterations in lung structure and function. Several longitudinal studies have demonstrated that individuals born preterm with a history of BPD frequently develop persistent respiratory symptoms, impaired lung function with persistent obstruction and increased susceptibility to respiratory infections as well as abnormal thoracic imaging throughout childhood and adulthood [1, 24, 25, 39–43]. Long-term lung function impairments may contribute to an increased risk of early development of chronic obstructive pulmonary disease later in life [42]. Given the scarcity of treatment options [38], early identification of at-risk infants is crucial. In the present study, infants with BPD exhibited higher LCI values at 12 months’ corrected age, suggesting sustained ventilation inhomogeneity beyond the neonatal period. MBW has emerged as a sensitive method to detect early abnormalities in ventilation distribution and small airway function in young children [29]. In particular, LCI has been widely used in the assessment of early lung disease in conditions such as cystic fibrosis [44]. However, data on MBW in preterm infants with history of BPD remain limited and heterogeneous [26, 32, 33, 45, 46]. While several studies have reported increased LCI values in older children [33, 47] and young adults [43] with a history of BPD, other investigations performed in the neonatal period did not observe clear differences between infants with and without BPD [32, 48]. One possible explanation for these discrepant findings may be differences in the timing of lung function measurements. For example, the study by Latzin et al. assessed lung function at a postconceptional age of approximately 44 weeks, whereas our measurements were performed at 12 months’ corrected age. It is therefore conceivable that abnormalities in ventilation distribution become more apparent later during infancy when lung size, breathing pattern, and postnatal airway and alveolar development have progressed, whereas measurements performed around term-equivalent age may not yet capture these functional abnormalities. In contrast to the LCI, the weight corrected tidal volume and minute ventilation breathing did not differ in infants with and without BPD at the corrected age of 12 months. Previous studies performed around term-equivalent age similarly reported no significant differences in tidal volume after adjustment for body weight in infants with and without BPD [32]. Another longitudinal study reporting on tidal volume and minute ventilation measured at 15 months corrected age also did not find a difference between infants with and without BPD when parameters were corrected for weight [48]. Importantly, tidal volume and minute ventilation reflect different physiological aspects of respiratory function than MBW-derived parameters such as LCI. While LCI is considered sensitive to ventilation inhomogeneity likely reflecting structural abnormalities of the peripheral airways, regional differences in compliance and resistance or air trapping, weight adjusted tidal breathing parameters may remain relatively preserved despite underlying alterations in lung structure.

Another important observation in our study was the positive correlation between LCI and the duration of neonatal respiratory support, including CPAP ventilation and oxygen supplementation. These findings are consistent with the concept that the severity and duration of neonatal lung injury may influence long-term respiratory outcomes. Interestingly, we also observed an association between LCI and the duration of intravenous antibiotic treatment during the neonatal period. Further exploratory analysis showed that infants with 2 or more episodes of LOS, possibly a sign of sustained inflammation, and patients that required treatment with broad-spectrum antibiotics had higher LCI values. Other studies have also reported associations of sustained inflammation with adverse long term outcomes, such as retinopathy of prematurity [49] or impaired long-term motor development [50]. Antibiotic exposure in early life has previously been linked to alterations of the developing microbiome and may influence immune and lung development. Data from the German Neonatal Network showed that multiple episodes of perinatal antibiotic exposure were associated with a reduced FEV1 z-score at 5 to 7 years of age [51]. However, antibiotic treatment may also reflect the severity of neonatal illness rather than representing a direct causal factor. Therefore, this association should be interpreted with caution and warrants further investigation in larger longitudinal studies.

Respiratory morbidity during the first year of life was also associated with impaired lung function in our cohort. BPD patients with high numbers of upper respiratory tract infections had higher LCI values compared to BPD patients with lower number of upper respiratory tract infections and infants without BPD. These findings need to be interpreted with caution, due to the limited sample size and the exploratory nature of our study. Infants who required hospital readmission due to respiratory symptoms showed higher LCI values at 12 months’ corrected age. This observation supports previous reports indicating that preterm infants with impaired lung function are particularly vulnerable to respiratory infections during early childhood [1, 24, 39–41]. Whether early lung function abnormalities contribute to increased susceptibility to infections or whether recurrent infections further impair lung development remains to be determined.

The main strengths of our study include the detailed clinical characterization of a well-defined cohort of preterm infants and the use of MBW measurements to assess lung function during the first year of life. In addition, lung function testing was performed during spontaneous sleep without the need for sedation, which may facilitate the application of this method in future follow-up studies in a research setting. Our study shows, that lung function testing during spontaneous sleep at 12 months corrected age requires a highly standardized setting, experienced personnel and close collaboration with parents. The limitations of our study are inherent to single-center design and moderate sample size of infants with successful MBW measurements. The feasibility rate of successful MBW measurement was 40% in our study cohort. Most previous MBW studies at this age used chloral hydrate sedation for MBW measurements. One South African birth cohort study reported a higher success rate of 70% in MBW measurements of healthy term-born infants at one year of age during spontaneous sleep [52], whereas lower feasibility (53%) has been described in infants of 12 months with acute respiratory disease such as bronchiolitis [53]. To our knowledge, this is the first study reporting MBW measurements in premature infants during spontaneous sleep at the corrected age of 12 months. Preterm infants may exhibit altered sleep architecture and less stable sleep states compared with term-born infants, which could affect the feasibility of lung function testing during natural sleep. The moderate sample size of infants with successful MBW measurements limits generalizability and does not account for potential confounding factors. Local treatment strategies in the neonatal phase could also influence the diagnosis of BPD and the LCI. In addition, respiratory morbidity during the first year of life was assessed based on parental reports and may therefore be subject to recall bias; however, questionnaires were completed jointly by parents and study physicians in order to improve accuracy. Finally, although we identified associations between LCI and several neonatal factors, the study does not allow conclusions regarding causality.

In summary, MBW measurements at 12 months’ corrected age are feasible in a subset of preterm infants in a specialized research setting, but limited feasibility currently restricts their broader clinical implementation. MBW-derived parameters indicated persistent ventilation inhomogeneity in preterm infants with a history of BPD, and LCI was associated with neonatal respiratory support and respiratory morbidity during the first year of life. These findings suggest that LCI may reflect early lung function impairment in this population. Larger longitudinal studies are required to determine whether MBW-derived parameters can predict long-term pulmonary outcomes, including spirometry-based lung function outcomes at school age, and whether they may help guide early preventive interventions in preterm infants.

## Supplementary Information

Supplementary Material 1.

## Data Availability

The data that support the findings of this study are not openly available due to reasons of sensitivity and are available from the corresponding author upon reasonable request.
