# Supplementary material for: Multiple-breath washout at 12 months of age reveals lung function impairments in preterm infants with bronchopulmonary dysplasia
Source: Mol Cell Pediatr. 2026 Jul 28;13:40. doi: 10.1186/s40348-026-00248-x (PMC13415711; doi:10.1186/s40348-026-00248-x)
Supplement: Supplementary file 1 — Supplementary Material 1. [file 40348_2026_248_MOESM1_ESM.docx]

**Supplemental Figure 1: Selection of items of the parental questionnaire used at the 12 months study visit. English translation.**

**Supplemental Table 1: Subgroup analysis: Clinical characteristics of VLBW infants with successful MBW measurements stratified by BPD status**

|  | VLBW, no BPD, n = 17 | BPD, n = 15 | p-value |
| --- | --- | --- | --- |
| GA (w) | 29.3 ± 1.4 | 26.2 ±1.7 | <0.001 |
| BW (g) | 1236 ± 241 | 845 ± 319 | <0.001 |
| Sex - male | 7 (42%) | 10 (67%) | 0.149 |
| Caesarean section | 14 (83 %) | 13 (87%) | 0.737 |
| Surfactant | 12 (71 %) | 14 (93%) | 0.100 |
| Mechanical ventilation (d) | 0.2 ± 0.7 | 7.8 ± 7.65 | 0.002 |
| CPAP (d) | 27.7 ± 14.3 | 71.3 ± 31. | <0.001 |
| O2 (d) | 7.2 ± 9.1 | 69.47 ± 31.4 | <0.001 |
| EOS | 8 (47%) | 6 (40%) | 0.688 |
| LOS | 0 | 10 (67%) | <0.001 |
| ATB (d) | 10.7 ± 5.8 | 58.8 ± 49.2 | 0.002 |
| URTI (#) | 3.9 ± 3.2 | 2.9 ± 1.8 | 0.313 |
| Bronchitis (#) | 2.7 ± 5.0 | 0.9 ± 1.4 | 0.215 |
| Hospital re-admission^§^ | 0 | 5 (35%) | 0.010 |
| Body length 12M (cm) | 74.1± 2.6 | 73.3 ± 4.9 | 0.584 |
| Body weight 12M (kg) | 9.47 ± 1.40 | 8.84 ± 1.28 | 0.095 |

Data are described as mean (standard deviation) or n (%).Abbreviations: VLBW: very low birth weight, GA (w) = gestational age in weeks, BW (g) = birth weight (grams), CPAP (d): duration of CPAP ventilation in days, O2 (d)= duration of oxygen supplementation in days, EOS = early onset sepsis, LOS = late onset sepsis (clinically diagnosed and blood culture positive), ATB (d) = duration of intravenous antibiotic treatment in days, URTI (#): number of upper respiratory tract infections, §=hospital re-admission due to respiratory symptoms, 12M= 12 months.

**Supplemental Table 2: Subgroup analysis: TBFVL and MBW results in preterm infants in VLBW infants stratified by BPD status.**

| TBFVL, n= 44 | VLBW, no BPD  n = 25 | BPD  n =19 | p-value |
| --- | --- | --- | --- |
| RR (/min) | 26 ± 5 | 28 ± 9 | 0.463 |
| MV (ml/min) | 2575 ± 390 | 2420 ± 363 | 0.186 |
| MV/weight (ml/min*kg) | 282 ± 41 | 279 ± 38 | 0.790 |
| VT (ml) | 98 ± 16 | 91 ± 21 | 0.165 |
| VT/weight (ml/kg) | 10 ± 2.0 | 10 ± 2.1 | 0.448 |

| MBW, n= 33 | VLBW, no BPD  n = 17 | BPD  n = 15 | p-value |
| --- | --- | --- | --- |
| FRC (l) | 0.19 ± 0.05 | 0.23 ± 0.06 | 0.043 |
| VT/FRC | 0.58 ± 0.16 | 0.40 ± 0.12 | 0.001 |
| LCI | 6.4 ± 0.51 | 7.0 ± 0.66 | 0.011 |

Data are described as mean ± standard deviation or n (%). Abbreviations: VLBW: very low birth weight, BPD = bronchopulmonary dysplasia, RR = respiratory rate, MV = minute volume, VT = tidal volume, FRC = functional residual capacity, LCI = lung clearance index, t-test.

**Supplemental Table 3: Clinical characteristics of preterm infants stratified by BPD status and number of upper respiratory tract infections (URTIs) and successful MBW measurements**

|  | URTIs <4  No BPD n=18 | URTIs ≥4  No BPD  n=12 | p-value * | URTIs <4  BPD  n=10 | URTIs ≥4  BPD  n=5 | p-value ** |
| --- | --- | --- | --- | --- | --- | --- |
| GA (w) | 31.9 ± 2.6 | 30.0 ± 2.0 | 0.047 | 26.4 ± 1.1 | 25.7 ± 2.6 | 0.458 |
| BW (g) | 1799 ±752 | 1299 ± 363 | 0.063 | 826 ± 262 | 882 ± 445 | 0.761 |
| Sex - male | 12 (67%) | 5 (42%) | 0.176 | 5 (50%) | 5 (100%) | 0.053 |
| Caesarean section | 12 (67%) | 11 (92%) | 0.113 | 10 (100%) | 3 (60%) | 0.032 |
| Surfactant | 7 (39%) | 6 (5%) | 0.638 | 9 (90%) | 5 (100%) | 0.464 |
| Mechanical ventilation (d) | 0.2 ± 0.7 | 0.0 ± 0.0 | 0.163 | 7.4 ±7.5 | 8.6 ± 8.6 | 0.786 |
| CPAP (d) | 15.2 ± 15.5 | 20.6 ± 17.3 | 0.384 | 55.5 ± 11.9 | 103.2 ± 33.8 | 0.032 |
| O2 (d) | 5.4 ± 8.5 | 4.8 ± 6.2 | 0.833 | 58.4 ± 25.3 | 91.6 ± 33 | 0.049 |
| EOS | 5 (28%) | 4 (33%) | 0.745 | 3 (30%) | 3 (60%) | 0.264 |
| LOS | 1 (6%) | 2 (17%) | 0.320 | 5 (50%) | 5 (100%) | 0.053 |
| ATB (d) | 8.8 ± 5.8 | 13.5 ± 10.1 | 0.113 | 74.1 ± 4.7 | 71.6 ± 5.6 | 0.216 |
| URTI (#) | 2.1 ± 0.6 | 6.3 ± 4.1 | 0.002 | 1.9 ± 1.0 | 5.0 ± 1.0 | <0.001 |
| Bronchitis (#) | 0.3 ± 0.5 | 3.7 ± 5.8 | 0.036 | 0.3 ± 0.5 | 2.2 ±1.8 | 0.038 |
| Hospital re-admission^§^ | 0 | 0 | n.a. | 1 (10%) | 4 (80%) | 0.007 |
| Body length 12M (cm) | 74.9 ± 3.6 | 76.0 ± 2.6 | 0.614 | 74.1 ± 4.6 | 71.6 ± 5.6 | 0.374 |
| Body weight 12M (kg) | 9.64 ± 1.24 | 9.70 ± 1.51 | 0.909 | 9.05 ± 1.23 | 8.41 ± 1.42 | 0.377 |
| Severe BPD | n.a. | n.a. | n.a. | 3 (30%) | 5 (100%) | 0.010 |

Data are described as mean (standard deviation) or n (%).Abbreviations: GA (w) = gestational age in weeks, BW (g) = birth weight (grams), CPAP (d): duration of CPAP ventilation in days, O2 (d)= duration of oxygen supplementation in days, EOS = early onset sepsis, LOS = late onset sepsis (clinically diagnosed and blood culture positive), ATB (d) = duration of intravenous antibiotic treatment in days, URTI (#): number of upper respiratory tract infections, §=hospital re-admission due to respiratory symptoms, 12M= 12 months. *comparison of infants with less than 4 URTIs to infants with 4 or more URTI within the subgroup of patients without BPD. **comparison of BPD patients with less than 4 URTIs to BPD patients with 4 or more URTIs.

**Supplemental Table 4: Clinical characteristics of preterm infants with and without hospital re-admission due to respiratory symptoms**

|  | No hospital re-admission  N=40 | Hospital re-admission  N=5 | p-value |
| --- | --- | --- | --- |
| GA (w) | 29.9 ± 3.1 | 25.8 ± 2.6 | 0.005 |
| BW (g) | 1440 ± 672 | 844 ± 430 | 0.061 |
| Sex - male | 23 (58%) | 4 (80%) | 0.333 |
| Caesarean section | 33 (83%) | 3 (60%) | 0.236 |
| Surfactant | 22 (55%) | 5 (100%) | 0.059 |
| Mechanical ventilation (d) | 2.0 ± 4.8 | 8.0 ± 9.3 | 0.224 |
| CPAP (d) | 27.4 ± 23.6 | 99.0 ± 37.8 | <0.001 |
| O2 (d) | 18.6 ± 27.1 | 91.0 ± 34.1 | <0.001 |
| EOS | 12 (30%) | 3 (60%) | 0.180 |
| LOS | 8 (20%) | 5 (100%) | <0.001 |
| ATB (d) | 18.5 ± 17.7 | 92.4 ± 75 | 0.092 |
| URTI (#) | 3.43 ± 3.0 | 4.2 ±2.1 | 0.580 |
| Bronchitis (#) | 1.43 ± 3.5 | 1.4 ± 0.9 | 0.988 |
| Body length 12M (cm) | 75.1 ± 3.5 | 70.4 ± 5.3 | 0.011 |
| Body weight 12M (kg) | 9.50 ±1.32 | 8.44 ± 1.45 | 0.100 |
| BPD | 10 (25%) | 5 (100%) | <0.001 |
| Severe BPD | 4 (10%) | 4 (80%) | <0.001 |

Data are described as mean (standard deviation) or n (%).Abbreviations: GA (w) = gestational age in weeks, BW (g) = birth weight (grams), CPAP (d): duration of CPAP ventilation in days, O2 (d)= duration of oxygen supplementation in days, EOS = early onset sepsis, LOS = late onset sepsis (clinically diagnosed and blood culture positive), ATB (d) = duration of intravenous antibiotic treatment in days, URTI (#): number of upper respiratory tract infections, §=hospital re-admission due to respiratory symptoms, 12M= 12 months.
